# Impact of HIV-1 viral subtype on disease progression and response to antiretroviral therapy

**DOI:** 10.1186/1758-2652-13-4

**Published:** 2010-02-03

**Authors:** Philippa J Easterbrook, Mel Smith, Jane Mullen, Siobhan O'Shea, Ian Chrystie, Annemiek de Ruiter, Iain D Tatt, Anna Maria Geretti, Mark Zuckerman

**Affiliations:** 1 Department of HIV/GU Medicine, King's College London School of Medicine at Guy's, King's College and St Thomas' hospitals, Weston Education Centre, 10 Cutcombe Road, London, SE5 9RJ, UK; 2 Health Protection Agency London, London South Specialist Virology Centre, Bessemer Road, London, SE5 9RS, UK; 3 Department of Virology and HIV/GU Medicine, St Thomas' Hospital, Westminster Bridge Road, London, SE1 7EH, UK; 4 Virus Reference Department, Health Protection Agency, Centre for Infections, 61 Colindale Avenue, London, NW9 5HT, UK; 5 Department of Virology, Royal Free Hospital and Royal Free and University College Medical School, Pond Street, London, NW3 2QG, UK; 6 Pharmaceuticals Division, Hofffman-La Roche AG, Basel, Switzerland

## Abstract

**Background:**

Our intention was to compare the rate of immunological progression prior to antiretroviral therapy (ART) and the virological response to ART in patients infected with subtype B and four non-B HIV-1 subtypes (A, C, D and the circulating recombinant form, CRF02-AG) in an ethnically diverse population of HIV-1-infected patients in south London.

**Methods:**

A random sample of 861 HIV-1-infected patients attending HIV clinics at King's and St Thomas' hospitals' were subtyped using an in-house enzyme-linked immunoassay and *env *sequencing. Subtypes were compared on the rate of CD4 cell decline using a multi-level random effects model. Virological response to ART was compared using the time to virological suppression (< 400 copies/ml) and rate of virological rebound (> 400 copies/ml) following initial suppression.

**Results:**

Complete subtype and epidemiological data were available for 679 patients, of whom 357 (52.6%) were white and 230 (33.9%) were black African. Subtype B (n = 394) accounted for the majority of infections, followed by subtypes C (n = 125), A (n = 84), D (n = 51) and CRF02-AG (n = 25). There were no significant differences in rate of CD4 cell decline, initial response to highly active antiretroviral therapy and subsequent rate of virological rebound for subtypes B, A, C and CRF02-AG. However, a statistically significant four-fold faster rate of CD4 decline (after adjustment for gender, ethnicity and baseline CD4 count) was observed for subtype D. In addition, subtype D infections showed a higher rate of virological rebound at six months (70%) compared with subtypes B (45%, p = 0.02), A (35%, p = 0.004) and C (34%, p = 0.01)

**Conclusions:**

This is the first study from an industrialized country to show a faster CD4 cell decline and higher rate of subsequent virological failure with subtype D infection. Further studies are needed to identify the molecular mechanisms responsible for the greater virulence of subtype D.

## Introduction

The world-wide HIV epidemic has been characterized by increasing genetic diversity, with multiple distinct viral subtypes, as well as sub-subtypes, and circulating recombinant forms (CRFs) [1-3]. At present, specific subtypes and CRFs are found more frequently in certain countries or regions of the world. Globally, the main variants are subtype C, which predominates in south and east Africa, followed by subtype A and the recombinant form CRF02-AG in west and west-central Africa.

Although subtype B dominates in North America, western Europe and Australia, the recent epidemiology of HIV-1 infection in the UK and many western European countries has been characterized by a marked increase in the prevalence of non-B subtypes and several CRFs [4-9]. In the UK, the number of new diagnoses due to heterosexually acquired infection has risen almost four-fold since 1996. The majority (> 95%) of these infections are likely to have been acquired abroad, mainly in sub-Saharan Africa but also in the Caribbean basin and Asia, a fact that is reflected in the heterogeneous pattern of subtypes in the heterosexually acquired HIV-1-infected population in the UK [10]. There is also evidence for onward transmission of these non-B HIV-1 strains within the UK [11].

Given the increasing subtype diversity in various sub-populations, the potential for the emergence of novel genetic variants, and the increasing availability of antiretroviral therapy (ART) worldwide, it has become even more important to establish the clinical implications of subtype variation [12-14]. Limitations of previous studies on the impact of subtype on disease progression have included a small sample size, use of seroprevalent cohort data, and the tendency to analyze non-B subtypes as a single group [15-25].

However, several recent studies from sub-Saharan Africa have found higher rates of disease progression in individuals infected with subtype D virus [16,22-25]. There remains still very limited data on HIV-1 subtype differences in the response to ART [26-30]. The main objectives of our study were to compare the rate of disease progression, based on rate of CD4 decline prior to ART, and the initial and subsequent virological response to ART in an ethnically diverse population in south London infected with subtype B and the most common non-B HIV-1 subtypes (A, C, D and CRF02-AG).

## Methods

### Study population

King's College Hospital and St Thomas' Hospital HIV clinics are based in the inner London boroughs of Lambeth, Southwark and Lewisham. In addition to containing a large migrant population from sub-Saharan Africa and a significant black Caribbean community, these areas have the highest rate of new HIV diagnoses in the UK. The two clinics care for a heterogeneous population of almost 3000 HIV-1-infected patients, a large proportion of whom originate from sub-Saharan Africa. We selected an approximately 50% random sample of 861 patients (456 from King's College Hospital and 405 from St Thomas' Hospital) based on all adult (≥ 18 years) HIV-1-infected patients who had attended the HIV clinic at either site over a one-year period between May 1999 and May 2000.

### Data collection

HIV-1-infected patients receiving antiretroviral therapy are seen routinely at three- to four-month intervals for clinical evaluation, monitoring of CD4 count, viral load, haematology and biochemistry. Those not on antiretroviral therapy are reviewed every three to six months. The criteria for initiation of ART are the presence of a CD4 cell count of < 350 cells/mm^3 ^or the presence of symptomatic HIV disease. CD4 cell counts were determined with a FACScount apparatus (Becton Dickinson) in freshly collected whole blood.

The local HIV clinic databases and the patients' medical records were used to obtain demographic data (ethnic origin, country of birth, gender, HIV transmission risk group, and age at HIV diagnosis), and clinical and laboratory data (clinical stage at presentation, CD4 count and viral load within three months of HIV diagnosis, together with a longitudinal record from initial diagnosis of all clinical events, serial CD4 cell counts and viral load, and all ART drug prescriptions). Ethnic group was based on self-reported ethnicity on the clinic registration form and country of birth.

At one of the two clinic sites (King's College Hospital), adherence to the antiretroviral drug regimen is assessed routinely at each clinic visit by documenting the number of times a specific drug dose had been missed, as well as the number of times it had been taken more than two hours late over the preceding 30-day period.

### Laboratory methods

HIV-1 serotyping was performed on the first available plasma sample after HIV diagnosis from all patients using an in-house enzyme linked immunoassay (EIA) directed against peptide antigens representative of the V3 region of the outer envelope glycoprotein, gp120 [31], and was used to discriminate between B and non-B subtypes [32]. *Env *sequencing was performed to assign a subtype to samples identified as non-B-using EIA (n = 124) and in samples with mixed reactivity (n = 19) or non-reactivity (n = 71) on serology. In addition, *env *sequencing was performed to validate EIA-determined subtype B infections in all black Africans, the majority of black Caribbeans, and in a sample of white patients with a subtype B infection (n = 30); previous studies have shown that serological typing can discriminate between B and non-B subtypes with a high degree of specificity in populations with predominantly subtype B infections [31].

*Env *sequencing was performed at the Dulwich Health Protection Agency (HPA) or at the HPA Sexually Transmitted and Blood Borne Virus Laboratory, Colindale, London. Samples processed at Dulwich HPA were extracted using QiAamp Viral RNA Mini Kits (Qiagen Ltd, Crawley, UK) according to the manufacturer's instructions. Amplification and cycle sequencing reactions were carried out as previously described [11]. Sequencing reactions (1.5 μL) were run on a Visible Genetics sequencing system under standard conditions using version 3.1.6 software. Samples handled at the HPA, Colindale, were extracted using a modified Boom method [32] and amplification was performed. Sequencing was performed using a CEQ 2000 XL DNA Analysis System capillary sequencer (Beckman Coulter, High Wycombe, UK) according to the manufacturer's instructions.

Samples were assigned a subtype by phylogenetic analysis of *env *gene alignments. HIV-1 reference sequences representative of group M subtypes (A-K), the most common CRFs, and groups O and N were obtained from the Los Alamos HIV sequence database http://www.hiv.lanl.gov/. Alignments of the study (where at least 240 base pairs [bp] of unambiguous sequence were available) and reference sequences were generated using the latest version of CLUSTAL W http://www.ebi.ac.uk/clustalw/ within Bioedit v4.8.5. The parameters of the optimal model of evolution were estimated using Modeltest v3.0 within the phylogenetic analysis package Phylogenetic Analysis Using Parsimony (Paup*), and neighbor-joining trees (bootstrapped × 1000) were generated. The tree topology was used to assign subtypes based on a high level of bootstrap support (> 70%) for each subtype or CRF cluster.

When a phylogenetically determined subtype was available, this was used to assign a definitive subtype. Two further subtyping methods were applied as a validation of the phylogenetic analysis results and to enable a subtype to be assigned to samples for which phylogenetic analysis was not possible: the National Center for Biotechnology Information Retrovirus Genotyping Tool (http://www.ncbi.nlm.nih.gov/projects/genotyping/ with a window size of 100 bp and an increment step of 50 bp; and the Basic Local Alignment Search Tool (BLAST 2.0) search tool at Los Alamos database http://www.hiv.lanl.gov/content/sequence/BASIC_BLAST/basic_blast.html.

### Algorithm for assignment of HIV-1 subtype

We developed a final algorithm to incorporate serology and genotypic subtype. Where an HIV-1 subtype was available by genotyping (n = 289; 221/345 of non-B subtypes; 68/407 of B subtypes), this was used to assign subtype. Where an HIV-1 sequence subtype was not available, as was the case with most B subtypes, an EIA-defined subtype B infection was assigned (n = 463; 339/407 B subtype; 124/345 non-B subtype). Because serology is unreliable for discriminating between B and non-B subtype in low subtype B prevalence populations such as black Africans [33], only subtype B infections in black Africans that had been confirmed by sequencing were included in the analysis.

### Statistical methods

We compared the demographic, clinical and laboratory characteristics at HIV diagnosis among patients infected with subtype B and the four main non-B subtypes (A, C, D and CRF02-AG), using chi-squared tests for categorical variables, and either Kruskal-Wallis or Wilcoxon rank-sum tests for continuous variables. We first compared subtype B with all non-B infections combined, and then conducted a series of pair-wise comparisons for the individual non-B subtypes.

We first compared the rate of CD4 cell decline prior to starting antiretroviral therapy in patients infected with B and non-B subtypes (A, C, D and CRF02-AG) based on their pre-therapy longitudinal CD4 cell count profiles. The rate of decline in square root transformed CD4 cell count for each subtype was estimated and compared using two-level random effects multiple regression models, and for four other variables (ethnicity, gender, HIV risk group and age). This model recognizes that the data are series of CD4 cell counts from the same individual over time, and allows each individual to have his or her own estimated intercept and rate of decline by introducing patient-specific elements (random effects). Multivariable regression analysis was used to examine for independent predictors of rate of CD4 decline using a backward elimination procedure. As the date of infection was not known in the majority of cases, all multivariate analyses were adjusted for the baseline CD4 cell count.

We also compared the time to virological suppression < 400 copies/ml following initiation of highly active antiretroviral therapy (HAART) and the time to virological rebound (based on two consecutive viral loads of > 400 copies/ml) after initial suppression, using Kaplan-Meier estimation. Log-rank tests were used to analyze pair-wise differences between subtypes, and all analyses were adjusted for percent adherence. Percent adherence was calculated for each patient visit by dividing the total number of missed or late doses by the total number of doses prescribed over a 30-day period, multiplied by 100. Percent adherence was analyzed as a binary variable (100% adherent vs. < 100% adherent); less than 100% adherence is associated with a significant reduction in attainment and maintenance of viral load suppression [34].

Univariate logistic regression analysis was used to examine the association between adherence and subtype, as well as other variables, including gender, risk groups and ethnicity, for each visit up to three visits. A generalized estimating equation (GEE) model for binary outcome with an exchangeable correlation matrix was also used to examine the relationship between adherence and subtype, incorporating up to three adherence assessments. Patients with more than one visit were used to examine changes in adherence overtime. A further analysis using a GEE model with an exchangeable correlation matrix was also performed for the subgroup of 94 patients with at least two adherence assessments. All data were analyzed using Stata 7.5 (Stata Corp., College Station, Texas, USA).

## Results

### Characteristics of 679 patients infected with B and non-B subtypes, A, C, D, and CRF02-AG

Of 861 patients, 182 patients were excluded from the analysis because their subtype could not be determined due to mixed reactivity or non reactivity (n = 109); unspecified non-B subtype (n = 16); less common non-B subtypes (n = 34); and incomplete epidemiological data (n = 23, 13 B, 4 A, and 6 C). Table S1, Additional file 1 shows the demographic, clinical and laboratory characteristics at HIV diagnosis of 679 patients infected with either subtype B (n = 394) or the four most common non-B subtypes - C (n = 125), A (n = 84), D (n = 51), or CRF02-AG (n = 25) - for whom complete epidemiological data was available. Of the 679 patients, 208 (30.6%) were female, 230 (33.9%) were black African, 301 (44.3%) had heterosexually acquired infection, and the median CD4 count and viral load at diagnosis was 315 (IQR = 164-481) and 12,400 (IQR = 1706-54,633), respectively. There were no statistically significant differences in the gender, ethnic group or risk group between the 679 and those excluded from the analysis.

Fifty-eight of 357 whites (16.2%) were infected with non-B subtypes (19 with subtype A, 16 with C, eight with D, and three with CRF02-AG). Of the 230 black Africans, only 11 (4.8%) were infected with B subtype, and the most common non-B subtypes were C (98, 42.6%), A (61, 26.5%), D (40, 17.4%), and CRF02-AG (20, 8.7%). Of the 51 black Caribbeans, 38 (74.5%) were infected with B subtypes, and 13 (25.5%) with non-B subtypes (seven with C, three with A, two with D, and one with CRF02-AG).

There were no statistically significant differences between the non-B subtypes in demographic characteristics or stage of disease at presentation. However, compared to those infected with any of the four non-B subtypes, patients with subtype B were more than twice as likely to be male (89.1% vs. 35.3% to 48%) (all p < 0.001), to be white (78.9% vs. 2.8% to 22.6%) (all p < 0.001), and to be homosexual or bisexual versus heterosexual (74.4% vs. 6.4% to 15.5%) (all p < 0.001). The median CD4 cell count at HIV diagnosis was significantly lower in patients infected with non-B versus B subtype: 331 (IQR = 196-501) cells/mm^3 ^versus 250 (IQR = 100-449) in subtype A (p = 0.02), 250 (IQR = 141-413) in subtype C (p = 0.01), 249 (IQR = 30-508) in subtype D (p = 0.04), and 297 (IQR = 113-386) with CRF02-AG (p = 0.06). There were no statistically significant differences in the median age or viral load at HIV diagnosis, or in the type of ART regimen between B and any of the non-B subtypes (A, C, D and CRF02-AG) on pair-wise comparisons.

### Rate of CD4 cell change prior to antiretroviral therapy

We analyzed 2778 CD4 cell counts in 627 patients, after exclusion of 52 patients who had only a baseline CD4 count available. A median of six serial CD4 cell counts were available in 627 patients prior to the initiation of any antiretroviral therapy (representing 77.4% of subtype B, 73.8% of subtype A, 76% of subtype C, 76.5% of subtype D, and 72% of CRF02-AG). The median six-monthly decline in the square root transformed CD4 cell count was -0.22 (95% CI, -0.29, -0.15) for subtype B and -0.27 (-0.37, -0.16) for all non-B subtypes combined. However, there were non-B subtype-specific differences in the rate of CD4 change: -0.22 (-0.40, -0.05) for subtype A; -0.21 (-0.40, -0.04) for subtype C; -0.80 (-1.08, -0.52) for subtype D; and -0.01 (-0.42, 0.40) for CRF02-AG.

There were no statistically significant differences in the rate of CD4 decline between B versus A (p = 0.24) or B versus C (p = 0.99). However, subtype D-infected patients had a four-fold more rapid rate of CD4 decline compared with subtypes B, A and C (unadjusted p values: B vs. D, p = 0.05; A vs. D, p = 0.002; C vs. D, p = 0.05; and CRF02-AG vs. D, p = 0.01). There was no association between rate of square root CD4 cell decline and ethnicity, gender risk group or age. The faster rate of CD4 decline in subtype D compared to other major subtypes remained significant after adjustment for ethnicity, gender and baseline CD4 cell count (B vs. D, p = 0.02; A vs. D, p = 0.002; C vs. D, p = 0.05; CRF02-AG vs. D, p = 0.01).

### Time to virological suppression < 400 copies/ml following initiation of HAART

**Figure 1:**
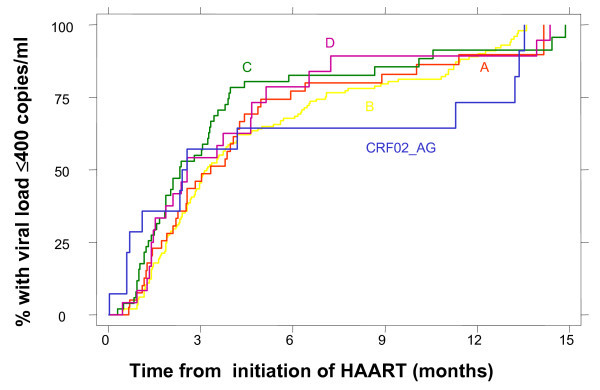
**Time to virological suppression < 400 copies/ml following initiation of HAART according to subtype**.

Overall, 374 of 679 study patients commenced antiretroviral therapy; 217 were subtype B, 46 were subtype A, 68 were subtype C, 29 were subtype D, and 14 were CRF02-AG. Of these, 141 received a protease inhibitor (PI) based combination, 109 a non-nucleoside reverse transcriptase (NNRTI) based regimen, 98 a triple nucleoside reverse transcriptase (NRTI) regimen, and 26 an NNRTI and PI combination. There were no differences in the type of regimen across subtypes, although a higher proportion of CRF02-AG patients received a PI-based regimen. We found no significant differences between subtypes B, A, C, D and CRF02-AG in the time to achieve viral load suppression (< 400 copies/ml) after initiation of HAART (see Figure 1).

The Kaplan-Meier estimates for the percentage achieving viral load suppression at six, nine and 12 months was, respectively: 66%, 78% and 88% for subtype B; 77%, 81% and 88% for subtype A; 82%, 85%, 92% for subtype C; 78%, 88% and 88% for subtype D; and 65%, 65% and 74% for CRF02-AG. Pair-wise comparisons of viral load suppression for subtypes found no statistically significant differences (B vs. A, p = 0.98; B vs. C, p = 0.24; B vs. D, p = 0.98; B vs. CRF02-AG, p = 0.40; A vs. C, p = 0.32; A vs. D, p = 0.81; A vs. CRF02-AG, p = 0.84; C vs. D, p = 0.75; C vs. CRF02-AG, p = 0.56; D vs. CRF02-AG, p = 1.00). The findings were similar after adjusting for important confounders, such as HIV risk group, baseline viral load and CD4 cell count, type of HAART regimen, and levels of adherence.

Of the 374 patients who received HAART, an adherence assessment was available for at least one visit in 148 patients (this was 66/217 (30.4%) for B; 11/46 (23.9%) for A; 25/68 (36.8%) for C; 15/29 (51.7%) for D; and 5/14 (35.7%) for CRFO2-AG), for two visits in 94 and three visits in 53 patients. In this subgroup, the percentage of patients with 100% adherence at the first, second and third adherence assessments across all subtypes was 73 (49.3%), 57 (60.6%), and 32 (60.4%), respectively. We found no statistically significant differences across subtypes in the percentage with 100% adherence at the three visits, and no association between 100% adherence and subtype (p > 0.5), ethnicity, gender or risk group. The findings were similar when the analysis was repeated using a GEE model with an exchangeable correlation matrix incorporating all three adherence assessments.

### Time to virological rebound following initial viral load suppression < 400 copies/ml

**Figure 2:**
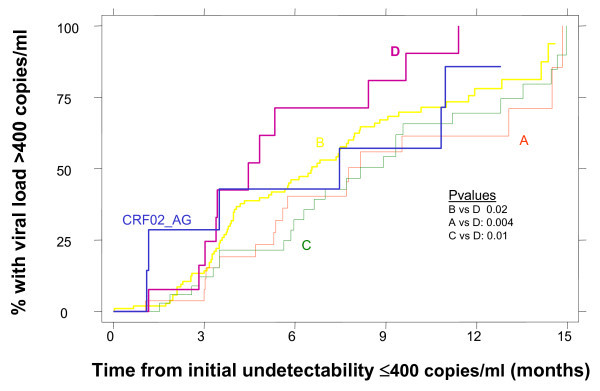
**Time to virological rebound following initial viral load suppression < 400 copies/ml according to subtype**.

Of the 133 subtype B patients who attained an initial viral load of < 400 copies/ml after initiation of HAART, 67 (45%) experienced subsequent viral load rebound by 12 months (defined as two consecutive counts of > 400 copies/ml) (see Figure 2). The percent of virological rebound at six months after initial viral load suppression was similar across subtypes B (45%), A (35%), C (34%) and CRF02-AG (44%), but significantly higher, at 70%, for subtype D (B vs. D, p = 0.02; A vs. D, p = 0.004; C vs. D, p = 0.01; D vs. CRF02-AG, p = 0.37).

In a Cox proportional hazards model, subtype D versus B infection and < 100% adherence were the only factors independently associated with an increased rate of virological rebound (Hazard Ratio [HR] = 2.14, 95% CI = 1.12-4.14, p = 0.02; HR = 1.32, 95% CI = 1.09-1.59, p = 0.004) after adjustment for baseline CD4 count and viral load, risk group and ethnicity. In contrast, subtype A versus B infection was associated with a reduced risk of viral rebound (HR = 0.67, 95% CI = 0.46-0.98, p = 0.039).

## Discussion

Our study was based on a large, well-characterized and ethnically diverse HIV-1-infected cohort in south London, half of whom were infected with the four main non-B subtypes (A, C, D and CRF02-AG), and with similar access to HIV care and monitoring and antiretroviral therapy. Although we found no clinically or statistically important differences in either the rate of immunological progression prior to antiretroviral therapy or in the initial virological response to antiretroviral therapy between subtype B and all non-B subtypes combined, certain non-B subtype-specific differences were observed. In particular, subtype D infection was associated with both a statistically significant four-fold faster rate of CD4 decline and a higher rate of virological rebound on ART compared with subtype B and the other main non-B subtypes, A and C.

We considered carefully whether the more rapid immunological progression in subtype D-infected patients than in patients with other subtypes could be explained by the shorter follow up, fewer CD4 cell count measurements and other differences in demographic characteristics. However, the faster rate of CD4 decline among subtype D patients remained statistically significant even after adjustment for all potentially important confounding factors, including gender, risk group, ethnic group and age. A further limitation of this and other studies on the impact of subtype on disease progression is that date of HIV infection was unknown. However, the baseline CD4 cell count, a surrogate for duration of infection, was similar across the non-B subtypes, but lower compared to B subtype infection [35], consistent with the more advanced disease at clinical presentation among black Africans who are more likely to be infected with non-B subtypes; this was adjusted for in the multivariate analysis.

Our study is the first from an industrialized country and including white patients infected with non-B subtypes to show a faster rate of disease progression with subtype D infection. This is consistent with the findings from five sub-Saharan African cohort studies [16,22-25]. In a study of 164 HIV-infected persons in Uganda (117 with incident infections), of which 65 were subtype A and 99 were subtype D, the relative hazard of AIDS-free survival was 1.39 (95% CI, 0.66-2.94, p = 0.39) for subtype D versus A. Those infected with subtype D and A/D recombinants also had a more rapid CD4 T cell decline, although this did not attain statistical significance [14]. In a further study from Uganda based on 1045 participants in a randomized controlled trial, subtype D was associated with a 1.29-fold increased risk of progression to death compared with subtype A [22]. Similarly, in the third Ugandan study based on 350 seroincident patients, the adjusted hazards for AIDS progression for subtype D was 2.13 (95% CI = 1.20-4.11) and for death 5.65 (95% CI = 1.37-23.4) relative to subtype D [25]. A further seroincident female cohort also found a two-fold higher mortality and rate of CD4 decline. These differences could not be explained by a higher viral load either at set point or over time [24].

Our findings of a similar rate of disease progression between subtypes B, A, C and CRF02-AG, are also consistent with those epidemiological studies that have examined for differences between these subtypes [15,17,19,20]. In one study, based on 126 individuals living in Sweden and infected with subtypes A, B, C and D [17], there were no statistically significant differences in the rate of CD4 cell decline or in the rate of clinical progression, although there was a small trend towards a faster rate of CD4 decline among subtype D-infected patients. In another cohort study of 91 Israeli men infected with subtype B and 77 Ethiopian immigrants infected with subtype C, the rate of change in CD4 percentage in the first two years following diagnosis was the same, -2.2%, for both groups [19]. In a cohort study of 336 patients from Cameroon and Senegal with approximately two years' follow up, there was no difference in survival, clinical disease progression and rate of CD4 decline between 207 patients with the CRF02-AG strain and the 128 patients infected with other strains (mainly A, n = 59; F, n = 17; and G, n = 15), followed by subtype C and D (each n = 10) [20]. Other studies from Thailand have compared subtypes B and E. In 130 seroconverters (103 with subtype E and 27 with subtype B), the viral load, CD4 and CD8 cell counts recorded one year after infection were similar in persons infected with either subtype, although the initial viral load at three months was three-fold higher among persons infected with subtype E [15].

Subtype-specific differences in virological and immunological characteristics may account for a faster rate of CD4 decline in subtype D-infected patients through an impact on viral load, viral tropism, syncytia formation and fitness, or immune response [3,12,14]. Although one study reported that subtype D-infected patients had higher viral loads during the course of infection than those with subtype A [36], we found no statistically significant differences in baseline or follow-up viral loads prior to initiation of antiretroviral therapy.

Viral genetic variation can influence phenotypic properties, such as cell tropism, co-receptor usage and the ability to form syncytia, although these properties have only recently been correlated with viral subtype. There is some evidence that subtype C uses only the CCR5 co-receptor, and has a preponderance of R5 or NSI viruses and a relative lack of X4 or SI viruses [37,38], while subtype D isolates tend to have a higher frequency of syncytium formation, CXCR4 (X4) coreceptor usage and rapidly replicating virus compared with other subtypes [39]. In a recent study of 31 Ugandan patients infected with subtype A and 35 with subtype D, there was a higher probability of X4 or dual tropic viruses in AIDS-free subtype D patients, which were also more replication competent. This suggests that an earlier switch to X4 virus with subtype D may explain the faster rate of CD4 decline and disease progression with subtype D [36].

In our study, HIV-1 subtype was determined by a combination of EIA and *env *sequencing, and an algorithm was devised to assign subtype. To clearly identify whether a sequence belonged to a subgroup representing a CRF within a certain subtype, phylogenetic analysis was done for each sequence individually. Significant misclassification in the assignment of subtype is unlikely as the majority of non-B subtype was assigned based on sequencing and phylogenetic analysis. The use of serologically defined subtype was mainly confined to subtype B infection among whites, as serotyping has been shown to be of good specificity for differentiating subtype B from non-B infections in populations predominantly infected with B subtype [31]. However, it is acknowledged that classification of subtype based on *env *or *gag *sequencing does not fully represent all aspects of the genetic variability of HIV, particularly the relationship to phenotypic properties, and that there may be other virological strain differences not captured by HIV-1 subtype.

We found no differences in the initial virological response to HAART, and in the proportion achieving a suppressed viral load six months after initiation of HAART according to subtype. This is consistent with the findings from three other cohort studies that compared the virological response to antiretroviral therapy based on HIV-1 subtype [26-29]. In a comparison of 265 European and 97 African patients (36% subtype D, 34% subtype C, and 13% subtype A), the initial virological and immunological responses were similar [26]. Similarly, in an analysis of 389 whites and 135 non-whites (mainly infected with non-B subtypes) in Denmark, and in 317 subtype B and 99 non-B-infected patients in France, there were no differences in the percentage who achieved viral load suppression of < 400 copies/ml [27,29]. However, neither of these studies performed any subtype-specific analyses across the different non-B subtypes, although in 113 children participating in the PENTA 5 clinical trial, HIV-1 subtype (16 A, 44 B, 47 C and 10 D) was not associated with virological outcomes at 24 and 48 weeks after initiation of HAART [28]. In a more recent analysis based on data from the UK Collaborative Group on HIV Drug Resistance and the UK Collaborative HIV Cohort Study [30], viral suppression occurred more rapidly in patients infected with subtype C (HR = 1.16, 95% CI = 1.01-1.33, p = 0.04) and subtype A (HR = 1.35, 95% CI = 1.04-1.74, p = 0.02) relative to subtype B infection, even after adjustment for lower baseline viral load in these subtypes.

Our study is the first to find a higher rate of rebound for subtype D (70% at 12 months compared to < 45% for all other subtypes), although this was based on only 11 patients with subtype D infection. Differences in antiretroviral compliance may also have contributed, although in our study, where adherence data was available on a subgroup of patients, we found no difference in levels of adherence according to either ethnic group or subtype.

In the much larger UK analysis [30], with overall significantly lower rates of viral rebound, there was a slight increased risk of viral load rebound from < 50 copies/ml only among patients infected with subtype C (and not subtype D) relative to subtype B, even after adjustment for probable non-adherence (adjusted hazards ratio, 1.40; 95% CI = 1.00-1.95, p = 0.05). In addition to the researchers' larger sample size, other important differences from our study were a different definition of viral load suppression and rebound, with overall lower rates of viral rebound.

There is increasing data on subtype-specific variations in susceptibility to antiretroviral drugs [12,14], with some well-documented differences in the resistance mutational patterns to specific drugs according to subtype [14,40-43]. Therefore, a further explanation for the higher rate of virological failure among patients with subtype D infection might be an increased propensity for the development of resistance to certain drugs. For example, recent data suggests that subtype D more easily develops resistance to non-nucleoside reverse-transcriptase inhibitors compared with subtype A infection [44-46], and the emergence of the D30N mutation to nelfinavir is favoured also in subtype D [40]. In the HIVNET 012 trial of single-dose nevirapine for prevention of mother to child transmission, nevirapine resistance mutations were present in 35.7% of subtype D compared to 19% with subtype A (p = 0.0035) [44]. This is further supported by a study from Argentina that demonstrated differential genetic barriers between subtypes leading to different rates of emergence of drug resistance-related mutations [47,48]. Importantly, we were unable to demonstrate any differences in the rate of virological failure for NNRTIs versus PIs across different subtypes.

## Conclusions

There is now a clear consensus that there is a similar rate of progression and response to HAART for subtype B and the non-B subtypes, A and C, but that subtype D is associated with a faster rate of disease progression. Our study is the first to report a higher rate of treatment failure for subtype D infection, but this will require confirmation in a larger cohort of patients infected with different subtypes and receiving antiretroviral therapy, with longitudinal data on adherence. Other detailed virological and immunological studies are needed to provide insights into the molecular mechanisms accounting for the apparent greater virulence of subtype D infection and potential implications for clinical management.

## Competing interests

The authors declare that they have no competing interests.

## Authors' contributions

PE coordinated the data collection and wrote the manuscript. MS and IT contributed to the preparation of the manuscript. MS, AMG, JM, SOS, IC, IT, NO and MZ performed the serotyping and *env *sequencing. AMR contributed clinical data.

All authors have read and approved the final manuscript.

## Supplementary Material

Additional file 1 **Table S1**. Characteristic of 679 patients infected with subtypes B, A, C, D and CRF02-AG. Click here for file
