# Supplementary material for: Impact of HIV-1 viral subtype on disease progression and response to antiretroviral therapy
Source: J Int AIDS Soc. 2010 Feb 3;13:4. doi: 10.1186/1758-2652-13-4 (PMC2827379; doi:10.1186/1758-2652-13-4)
Supplement: Additional file 1 — Table S1. Characteristic of 679 patients infected with subtypes B, A, C, D and CRF02-AG. [file 1758-2652-13-4-S1.DOC]

**Table S1: Characteristic of 679 patients infected with subtypes B, A, C, D and CRF02-AG**

|  | Subtypes | | | | | p-values for pair wise comparison of subtypes | | | | | |
| --- | --- | --- | --- | --- | --- | --- | --- | --- | --- | --- | --- |
|  | B  (n=394) | A  (n=84) | C  (n=125) | D  (n=51) | CRF02-AG  (n=25) | B  vs | B  vs | B  vs | A  vs | A  vs | C  vs |
|  | n (%) | n (%) | n (%) | n (%) | n (%) | A | C | D | C | D | D |
| **Gender**  Female  Male | 43 (10.9)  351 (89.1) | 45 (53.6)  39 (46.4) | 74 (59.2)  51 (40.8) | 33 (64.7)  18 (35.3) | 13 (52)  12 (48) | <0.001 | <0.001 | <0.001 | 0.42 | 0.20 | 0.50 |
| **Ethnicity**  White  Black African  Black Caribbean  Other or not known | 311 (78.7)  11 (2.8)  38 (9.6)  34 (8.6) | 19 (22.6)  61 (72.6)  3 (3.6)  1 (1.2) | 16 (12.8)  98 (78.4)  7 (5.6)  4 (3.2) | 8 (15.7)  40 (78.4)  2 (3.9)  1 (2.0) | 3 (12)  20 (80)  1 (4)  1 (4) | <0.001 | <0.001 | <0.001 | 0.33 | 0.79 | 0.93 |
| **Risk exposure**  Heterosexual  Homo/bisexual  Injecting drug user  Other or not known | 60 (15.2)  293 (74.4)  31 (7.9)  10 (2.5) | 68 (80.9)  13 (15.5)  1 (1.2)  2 (2.4) | 112 (89.6)  8 (6.4)  2 (1.6)  3 (2.4) | 41 (80.4)  7 (13.7)  0 (0)  3 (5.9) | 20 (80)  3 (12)  1 (4)  1 (4) | <0.001 | <0.001 | <0.001 | 0.18 | 0.23 | 0.19 |
| **At diagnosis**  Median age in years  (IQR) | 31.2  (26.9-36.4) | 32.9  (28-36.8) | 29.6  (25.3-34.9) | 30.9  (26.2-35.7) | 33.1  (27.6-36.4) | 0.41 | 0.10 | 0.62 | 0.07 | 0.31 | 0.55 |
| Median CD4 cell  count (x10^6^/l)  (IQR) | 331  (196-501) | 250  (100-449) | 250  (141-413) | 249  (30-508) | 297  (113-386) | 0.02 | 0.01 | 0.05 | 0.88 | 0.72 | 0.49 |
| Median viral load  (copies/l) (IQR) | 13,751  (2785-54,633) | 12,000  (1200-37,830) | 9726  (1181-51,720) | 13,917  (2861-112,447) | 23,106  (3974-45,615) | 0.25 | 0.11 | 0.66 | 0.85 | 0.26 | 0.22 |
| HAART regimen  PI  NNRTI  NRTI  NNRTI/PI | 79 (36.4%)  61(28.1%)  60(27.7%)  17(7.8%) | 13 (28.3%)  16 (34.8%)  14 (30.4%)  3 (6.5%) | 27(39.7%)  24(35.3%)  16(23.5%)  1 (1.5%) | 12 (41.4%)  7 (24.7%)  6 (20.7%)  4 (13.8%) | 10 (71.4%)  1 (7.1%)  2 (1.43%)  1 (7.1%) | 0.69 | 0.20 | 0.61 | 0.32 | 0.36 | 0.08 |

Note: p values for subtype B vs CRF02_AG were: gender, ethnicity and risk group (all<0.001). No other p values for pair wise comparison with CRF02_AG were found to be statistically significant.
